# Surgical strategy for an anomalous origin of the right coronary artery

**DOI:** 10.1186/1749-8090-8-S1-O142

**Published:** 2013-09-11

**Authors:** C Park, J Lee, Y Jun, C Choi, K Son, K Park

**Affiliations:** 1 Department of Thoracic and Cardiovascular Surgery, Gachon University, Gil Hospital, Incheon, Korea

## Background

According to the recent guidelines, revascularization is recommended (class I) for the symptomatic patients of an anomalous origin of the right coronary artery. However, the long-term outcome of several operations is not fantastic due to their pros and cons. We sought to determine whether multidetector computer tomography (MDCT) coronary angiography would be a good preoperative clue for surgical strategy.

## Methods

Between 2007 and 2013, 5 patients underwent unroofing or coronary bypass. For 2 patients, failure of unroofing inevitably led to bypass with saphenous vein during the operation. Thereafter, anatomical details of proximal right coronary artery (pRCA) were reviewed through the preoperative MDCT retrospectively.

## Results

All 5 patients (three females, age 13 - 63 years) survived asymptomatically during the follow-up period (30 - 81 months). The surgical intervention included unroofing in 2 patients (unroofing group), RITA bypass in 1 and bypass with saphenous vein in 2 (the native vessel was inevitably taken down: SV group). We measured the average diameters of the portion where RCA takes off from aorta (D1), diameters of the widest part of the pRCA (D2) and the thickness of aortic mural roof(H). D1 of the unroofing group (3.5 mm) was wider than that of the SV group (1.9 mm) and there was higher D1/D2 ratio in the unroofing group.

## Conclusion

The RCA diameter around the area where the RCA takes off from aorta can be an efficient clue to choosing the operative technique. It would be desirable to decide the operative modality after evaluating the anatomical feasibility through MDCT.

